# Biological Designer Self-Assembling Peptide Nanofiber Scaffolds Significantly Enhance Osteoblast Proliferation, Differentiation and 3-D Migration

**DOI:** 10.1371/journal.pone.0000190

**Published:** 2007-02-07

**Authors:** Akihiro Horii, Xiumei Wang, Fabrizio Gelain, Shuguang Zhang

**Affiliations:** 1 Center for Biomedical Engineering, Massachusetts Institute of Technology, Cambridge, Massachusetts, United States of America; 2 Olympus America Inc., Center Valley, Pennsylvania, United States of America; 3 Bioscience and Biotechnology Department, University of Milan-Bicocca, Milano, Italy; Center for Genomic Regulation, Spain

## Abstract

A class of self-assembling peptide nanofiber scaffolds has been shown to be an excellent biological material for 3-dimension cell culture and stimulating cell migration into the scaffold, as well as for repairing tissue defects in animals. We report here the development of several peptide nanofiber scaffolds designed specifically for osteoblasts. We designed one of the pure self-assembling peptide scaffolds RADA16-I through direct coupling to short biologically active motifs. The motifs included osteogenic growth peptide ALK (ALKRQGRTLYGF) bone-cell secreted-signal peptide, osteopontin cell adhesion motif DGR (DGRGDSVAYG) and 2-unit RGD binding sequence PGR (PRGDSGYRGDS). We made the new peptide scaffolds by mixing the pure RAD16 and designer-peptide solutions, and we examined the molecular integration of the mixed nanofiber scaffolds using AFM. Compared to pure RAD16 scaffold, we found that these designer peptide scaffolds significantly promoted mouse pre-osteoblast MC3T3-E1 cell proliferation. Moreover, alkaline phosphatase (ALP) activity and osteocalcin secretion, which are early and late markers for osteoblastic differentiation, were also significantly increased. We demonstrated that the designer, self-assembling peptide scaffolds promoted the proliferation and osteogenic differentiation of MC3T3-E1. Under the identical culture medium condition, confocal images unequivocally demonstrated that the designer PRG peptide scaffold stimulated cell migration into the 3-D scaffold. Our results suggest that these designer peptide scaffolds may be very useful for promoting bone tissue regeneration.

## Introduction

Regenerative medicine and tissue engineering require two complementary key ingredients: 1) biologically compatible scaffolds that can be readily adopted by the body system without harm, and 2) suitable cells including various stem cells or primary cells that effectively replace the damaged tissues without adverse consequences. However, it would be advantageous if one could apply suitable and active biological scaffolds to stimulate and promote cell differentiation, in addition to regenerating tissues without introducing foreign cells.

We previously reported the discovery and development of a class of self-assembling peptides made of only natural amino acids. This class of material peptides can undergo spontaneous assembly into well-ordered nanofibers and scaffolds, ∼10 nm in fiber diameter with pores between 5–200 nm and over 99% water content[1], [2]. These peptide scaffolds have three-dimensional nanofiber structures similar to the natural extracelluar matrices including collagen. They can readily be designed further to serve as a biomimic synthetic extracelluar matrix. These peptides have been used for the study of cell attachment, survival and proliferation[2]–[4], and injection into animals[3], [4]. When mixed together with other porous polymer scaffolds, the peptide scaffolds enhanced osteoblast growth and differentiation, suggesting possible application for bone tissue engineering[5].

The extracellular matrix is important, not only as a structural component for supporting cells, but also as a suitable microenvironment that influences cell-function. A number of short sequences in proteins located in the extracelluar matrix have recently been identified to play important roles for bone regeneration, including osteoblast proliferation, migration and differentiation[6]–[9]. These short peptides are secreted from cells locally [10] or in remote organs[11], and they have been shown to promote bone regeneration.

In the past few decades, a number of polymers and biopolymers have been attached with known biologically active peptide motifs from a variety of extracelluar matrix proteins to promote specific cellular responses[9], [12]–[14]. Modification of polymer scaffolds with cell attachment motif increased cell attachment and proliferation[9], [13], [14]. However, the progress in truly accelerated bone regeneration is still slow. A simple, inexpensive and universal bone regeneration treatment is still not available. Because of the increasing aging population in the world, bone disease will become more and more common, thus effective treatment for rapid bone regeneration becomes more and more urgent.

Recently a class of designer self-assembling peptide scaffolds has also been functionalized directly through solid-phase synthesis extension at the C-termini with short sequences including bone marrow homing motifs. These purely designer peptide scaffolds not only significantly enhanced adult mouse neural stem cell survival, proliferation, but also differentiation into neurons and glial cells [15]. Those studies encouraged us to specifically select a few relevant motifs to design new peptide scaffolds specifically for bone 3D cell culture to assess their activities. These active bone motifs are described below.

The osteogenic growth peptide OGP (ALKRQGRTLYGFGG) is a key factor in the mechanism of the systemic osteogenic response to local bone marrow injury. OGP level is increased in the serum in vivo during osteogenic reactions in the form of OGP-OGP binding protein complex. When it is applied to pre-osteoblast *in vitro*, OGP promotes bone cell proliferation and differentiation, which is indicated by increasing ALP Alkaline phosphatase activity[16]. When it is administered *in vivo*, OGP stimulates osteogenesis and hematopoiesis[16], [17].

Osteopontin, which has 264 to 301 amino acids depending on the species, is synthesized and phosphorylated in both osteoblasts and osteoclasts in bone. During odontogenesis, it is made by odontoblasts. Osteopontin regulates cell adhesion, migration, survival, NF-κB activity, NO synthesis and calcium crystal formation[18]. Osteopontin contains highly conserved functional domains, including calcium and heparin binding regions and a cell adhesion motif. The cell adhesion motif (DGRGDSVAYG) contains an RGD sequence and is highly conserved in many species.

Many investigators have studied the biological activities of the short peptide sequence Arg–Gly–Asp (RGD), which is present in fibronectin and many other extracellular matrix proteins. RGD sequence is important for cell adhesion[19]. Immobilization of RGD motif to polymers and alginate gel improves bone formation *in vitro* and *in vivo* [14].

Here we report the functionalization of the RADA16 self-assembling peptide scaffold RADA16 (AcN–RADARADARADARADA–CONH2) through direct solid phase synthesis extension at the C-termini with three short motifs from the osteogenic growth peptide OGP, the Osteopontin cell adhesion motif described above, and a designed 2-unit RGD binding sequence (PRGDSGYRGDS).

We showed that these designer peptide scaffolds significantly increased mouse preosteoblast MC3T3-E1 proliferation in comparison to pure RADA16 peptide scaffold. Moreover, the designer scaffolds promoted alkaline phosphatase (ALP) activity and Osteocalcin secretion, which suggests cell differentiation towards bone formation. These experiments showed that cell proliferation is enhanced using the mix of functional and pure RADA16 peptide scaffold in a concentration dependent manner. Most significantly, our observation show that the designer peptide scaffolds stimulated cell migration into the 3D scaffold, a key factor for the accelerated tissue regenerations. These results suggest that designer peptide scaffolds may be useful for bone regeneration and bone tissue engineering.

## Results

### Synthesis of new designer self-assembling peptides

We functionalized RADA16 with three biologically active motifs in order to develop a second generation of self-assembling peptide scaffolds to closer mimic extracellular matrices that enhance pre-osteoblast cell maintenance and function *in vitro*. These new peptide scaffolds were made by extending the original RADA16 at the C-terminus directly through solid phase synthesis with three short peptide motifs. They are an osteogenic growth peptide OGP (ALKRQGRTLYGFGG), Osteopontin cell adhesion motif DGR (DGRGDSVAYG) and a specifically designed 2 units of RGD sequence (PRGDSGYRGDS). These designer peptide sequences are listed in Table 1.

**Table 1:** Functionalized peptide scaffolds used in this study

Name	Sequences	Description
RADA16	Ac(RADA)_4_CONH_2_	Designed
ALK	Ac(RADA)_4_GGALKRQGRTLYGF-CONH_2_	From Osteogenic growth peptide
DGR	Ac(RADA)_4_GGDGRGDSVAYG-CONH_2_	From cell adhesion domain (Osteopontin)
PRG	Ac(RADA)_4_GPRGDSGYRGDS-CONH_2_	2-unit RGD motifs

The sequences are from N–>C. Ac  = acetylated N-termini, −CONH_2_ = amidated C-termini. The peptide motif sources from various protein origins. The 2-unit RGD motifs are purely molecular designed.

**Figure 1:**
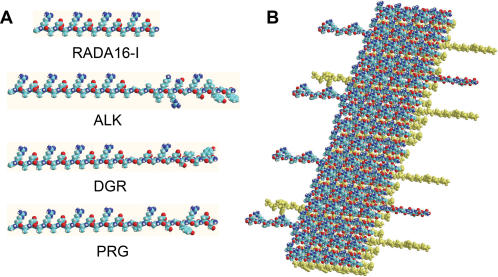
Molecular models of pure and designer peptide nanofibers. A) Models represent peptide RADA16, ALK, DGR and PRG from Table 1. B) Model representing a β-sheet double-tape of a self-assembling peptide nanofiber with PRG motif (4∶1). Note the sequences PRG extending out from the nanofiber double-tape.

The peptides were solubilized in water at a concentration of 10 mg/ml (1% w/v). DGR formed a hydrogel, but ALK and PRG remained as a non-viscous solution. When all peptide solutions are mixed 1∶1 with RADA16-I solution (1% w/v), they form highly viscous gel solutions. A molecular model representing the self-assembling peptide RADA16-I and the derived PRG are depicted in Fig. 1.

These designer peptide nanofiber scaffolds offer several advantages that include 1) easy design using known, biologically active motifs, 2) commercially custom-synthesized with mature solid phase peptide synthesis technology 3) selection of an extensive repertoire of biological active motifs detected in some extracellular matrix components and cell secreted peptide or protein.

### Structural study of the designer peptides

**Figure 2:**
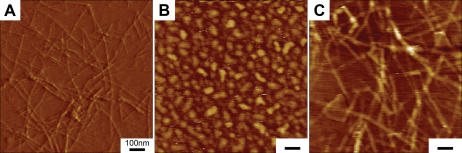
Tapping Mode AFM images of 1% (w/v) peptides solution of A) RADA16, B) PRG alone, C) PRG+RADA16 (1∶1). The bar represents 100 nm. The nanofiber formation is seen in A) RADA16 and C) PRG+RADA16 (1∶1). An increase in the fiber thickness in C) PRG+RADA16 (1∶1) (D = 29.5±3.1 nm) from A) RADA16 (D = 16.3±1.4 nm) was observed, which correlated to the width of the peptide fiber modeled in Fig. 1.

We studied nanofiber formation using Atomic Force Microscope (AFM). It has been previously reported that β-sheet structures of self-assembling peptides may be a prerequisite for self-assembly process into nanofibers[1], [2]. We used AFM (Tapping Mode) to analyze the formation of nanofibers because it allowed us to measure soft, fragile, and adhesive surfaces without damaging the samples. We examined 1% (w/v) peptide solution of RADA16, ALK, DGR, PRG and 1% (w/v) solution of ALK, DGR and PRG mixed with RADA16 at a ratio 1∶1. AFM images of RADA16, PRG separately and PRG-RADA16 composite are shown in Fig. 2. The nanofibers in aqueous solutions were observed in RADA16 and all RADA16 mixed solutions. These results were confirmed by visual inspection of increasing viscosity of the peptide scaffold solutions. We observed an increase in the fiber thickness in e) ALK+RADA16 (1∶1) (D = 35.5±2.9 nm), f) DGR+RADA16 (1∶1) (D = 26.6±2.4 nm) and g) PRG+RADA16-I (1∶1) (D = 29.5±3.1 nm) from a) RADA16 (D = 16.3±1.4 nm) as shown in Table 2. The molecular length of the peptides are RADA16, 5.9 nm; ALK, 10.7 nm; DGR, 10.0 nm; and PRG, 10.3 nm. The width of the peptide fiber thickness, based on the model shown in Fig. 1 are e) ALK+RADA16: 15.5 nm, f) DGR+RADA16: 14.1 nm) and g) PRG+RADA16: 14.7 nm). Table 2 shows the width of fiber considering the size effects of the AFM probe tip [20] and measured AFM. Given that the nanofibers were hydrated with water, the results are consistent. These results show that ALK and PRG solution cannot form nanofibers alone; instead, they may form a nanofiber scaffold when the individual ALK and PRG peptides are integrated into the RADA16 peptide nanofibers as proposed in the molecular model.

**Table 2:** Nanofiber width from molecular models and AFM measurements

Peptides	fiber width	AFM tip width)	(Peptide length w/size adjusted**)
a) RADA16	5.9 nm	12.1 nm	16.3±1.4 nm
b) ALK:	10.7 nm	16.9 nm	–
c) DGR:	10.0 nm	16.2 nm	–
d) PRG:	10.3 nm	16.5 nm	–
e) ALK+RADA16	15.5 nm*	21.7 nm	35.5±2.9 nm
f) DGR+RADA16	14.1 nm*	20.3 nm	26.6±2.4 nm
g) PRG+RADA16	14.7 nm*	20.9 nm	29.5±3.1 nm

* according to the molecular model proposed in Fig. 1

** based on the estimation W_adj_ = W_idth_+2(2R_t_H-H^2^)^1/2^, Rt = AFM tip size (∼10 nm), H: sample height (∼0.5 nm) [20]

### Cell growth on functionalized peptide matrices

**Figure 3:**
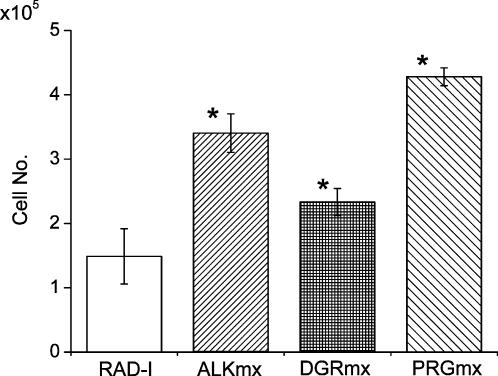
Cell numbers are calculated from DNA measurement on various scaffolds. MT3T3-E1 cells were cultured for 2 weeks on different scaffolds. RADA16∶RADA16 1% (w/v), ALKmx∶ALK 1% (w/v)+RADA16, DGRmx∶DGR 1% (w/v)+RADA16, PRGmx: PRG 1% (w/v)+RADA16 (all mix ratio is 1∶1). For peptide scaffolds containing active peptides, the cell proliferation rate is higher than that of pure RADA16. *p<0.01; suggesting it is significant against the number of cells grown in pure RADA16 scaffold.

We evaluated cell growth on functionalized peptide matrices by growing pre-osteoblast MC3T3E1 cells on the peptide scaffolds in culture inserts for two weeks. The cell numbers were calculated from DNA content extracted from the scaffolds. The cell numbers in each insert on the different scaffolds are shown in Fig. 3 after 2 weeks of continuous culturing. As expected, in the sequences contain RGD (DGR and PRG) cell proliferation was increased in comparison to the pure self-assembling peptide RADA16 alone, which does not contain RGD. ALK, DGR and PRG also showed statistically significant cell proliferation in comparison to RADA16 scaffold. These results reveal that the designer peptide scaffolds promote bone cell proliferation. These results are consistent with our previous report of adult mouse neural stem cell behaviors [15].

### Osteogenic differentiation of MC3T3 on functionalized peptide scaffolds

In order to evaluate the level of MC3T3-E1 differentiation, we used both ALP activity and osteocalcin concentration secreted in culture medium as markers, because ALP activity is considered an early marker for osteoblastic differentiation, but osteocalcin is considered a later differentiation marker related to bone biomineralization [14], [21]. MC3T3E1 cells on the peptide scaffolds were cultured in the tissue culture inserts for two weeks.

**Figure 4:**
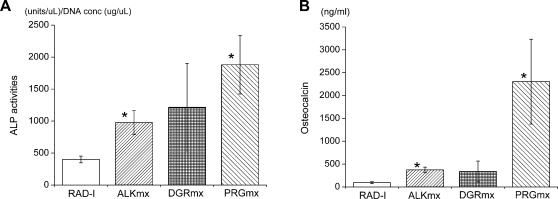
A) ALP activity normalized by DNA amount cultured on the different hydrogels for two weeks. RADA16∶RADA16 1% (w/v), ALKmx: ALK 1% (w/v)+RADA16-I, DGRmx∶DGR 1% (w/v)+RADA16, PRGmx∶PRG 1% (w/v)+RADA16 (all mixture ratio is 1∶1). ALK, DGR and PRG show the higher ALP activity compared to RADA16-I. *p<0.01 suggesting it is significant against the ALP activity in pure RADA16-I. B) Osteocalcin content secreted in culture medium after culturing on the different hydrogels for two weeks. RADA16∶RADA16 1% (w/v), ALKmx∶ALK 1%(w/v)+RADA16, DGRmx∶DGR 1%(w/v)+RADA16, PRGmx∶PRG 1% (w/v)+RADA16 (all mixture ratio is 1∶1). All modified peptides (ALK, DGR and PRG) show the higher osteocalcin contents compared to pure RADA16. PRG has a significantly higher concentration compared to the other scaffolds. *p<0.01 suggesting it is significant against Osteocalcin in RADA16 scaffold.

ALP activities were measured by ALP fluorometric assay using cell lysates. ALP activities were normalized by DNA measurement. Fig. 4A shows the ALP activities of MC3T3 cultured on the peptide scaffolds. Cells in ALK, DGR and PRG scaffolds show the higher ALP activity in comparison to the pure peptide scaffold RADA16. Cells in ALK and PRG scaffolds showed statistically significant higher ALP activity than cells cultured in RADA16 scaffolds.

We also measured the amount of Osteocalcin in the culture media using EIA method. Fig. 4B shows osteocalcin concentration secreted in tissue culture medium. Cells in all designer peptide scaffolds produced higher amounts of osteocalcin in comparison to cells in pure RADA16 scaffold. Cells in PRG scaffold produced significantly higher concentration of Osteocalcin in comparison to the other scaffolds.

**Figure 5:**
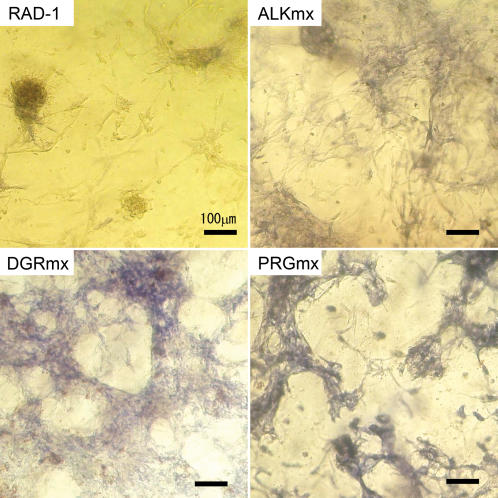
ALP Staining images after culturing on the different hydrogels for two weeks. The bar represents 100 µm. RAD-I∶RADA16 1% (w/v), ALKmx∶ALK 1% (w/v)+RADA16, DGRmx∶DGR 1% (w/v)+RAD, PRGmx∶PRG 1% (w/v)+RADA16 (all mixture ratio is 1∶1). The bluish color intensity correlates with the high ALP activity. RADA16 shows low cell adhesion to the hydrogel and the cells are aggregated. The cell attachment increases in DGR and PRG scaffolds were considered as a result of RGD cell attachment sequence. ALP, DGR and PRG showed higher ALP activities compared to RADA16-I, especially staining intensity of PRG.

We also measured the ALP production in the cells since it is known that ALP activity correlates with bone formation at particular stages. The results of ALP staining of MC3T3-E1 cells cultured on the scaffolds for two weeks are shown in Fig. 5. The bluish intensity is directly proportional to the ALP activity. However cells in RADA16 scaffold had lower cell adhesions and lower ALP activities. The cell attachment increase in DGR and PRG scaffolds were expected because they contain the RGD cell attachment sequence. Cells in ALP, DGR and PRG scaffolds again showed both higher cell attachment and higher ALP activities than cells in pure RADA16 scaffold. In particular, the ALP staining intensity of cells in the PRG scaffold is significant. The staining intensities of the ALP activities shown in Fig. 5 suggested the active bone cell differentiation.

### Effects of mix % of designer and RADA16-I scaffolds

**Figure 6:**
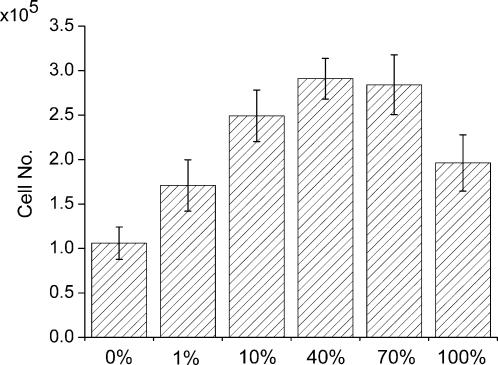
Cell numbers on the different scaffolds of different mix ratio of designer PRG and pure RADA16 scaffolds after 1-week culture. There is an increase in proliferation following the increase PRG % scaffold when increased from 0 to 40%. There was a decrease in cell proliferation at PRG 100% suggesting that there is an optimal ratio of PRG and RADA16 scaffolds.

In order to evaluate the effects of mix ratio of designer peptide and pure peptide scaffolds, we cultured MC3T3-E1 cells on the different scaffolds consisting of a different mix ratio of designer PRG and RADA16 scaffolds for 1 week. Fig. 6 shows the calculated cell numbers from DNA content measured in the scaffolds. When PRG scaffold was increased from 0 to 40%, there was a ∼3-fold increase of cell proliferation as determined by the DNA concentration assay of cells from PRG scaffold. Nevertheless, there was drop in cell proliferation at 100% PRG scaffold; this result suggests that 40% may be an optimal mix ratio of PRG and RADA16 scaffold for bone cell proliferation.

**Figure 7:**
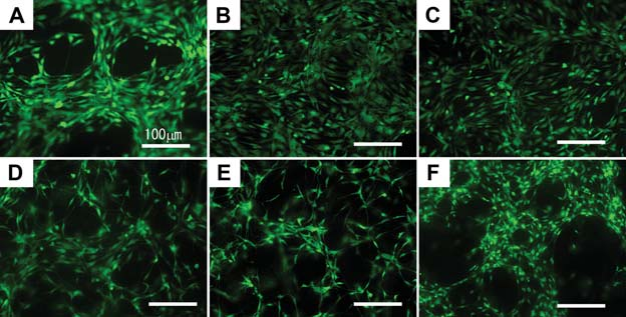
Cell morphology on the different scaffolds of various mix ratios of RADA16 1% (w/v) and PRG 1% (w/v) using calcein-AM staining. The bar represents 100 µm. A) RADA16 100%∶PRG 0%, B) PRG 1%, C) PRG 10%, D) PRG 40%, E) PRG 70%, F) PRG 100% (RADA16: 0%). B) PRG: 1% shows uniform cell distribution compared to (A) 0%, which shows the increase of cell attachment. There is significant morphological difference in (C) 10% and (D) 40%, as seen by a change in cell shape from elongated form to asteroid form.

The cell morphology was also examined on the different scaffolds of different mix ratio, using calcein-AM staining, which stains whole living cells (Fig. 7). The cells in 1% PRG scaffold showed different cell distribution in comparison to pure scaffold (0%). This observations reveal that even PRG as low as 1% is effective for increasing cell adhesion to the scaffold. There is significant morphological difference in Fig. 7C (10%) and Fig. 7D (40%): the cell shape was changed from flat into spindle-shaped. In Fig. 7 C, cells were on the surface of the scaffold, but in Fig. 7D, cells spontaneously migrated into 3-D scaffold.

### Cell migration into designer 3D scaffold but not pure RADA16 scaffold

**Figure 8:**
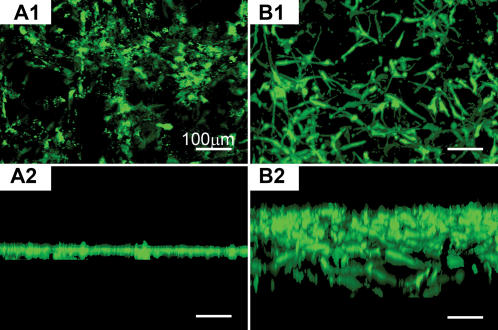
Reconstructed image of 3-D confocal microscope image of culturing on the different scaffolds consisting of different mix ratio of RADA16 1% (w/v) and PRG 1% (w/v) using calcein-AM staining. The bar represents 100 µm. A1 and A2: PRG 10% and B1&B2: PRG 70%. A1 and B1 are vertical view and A2 and B2 are horizontal view. In the case of 10% PRG scaffold, the cells were attached on the surface of the scaffold whereas the cells were migrated into the scaffold in the case of 70% PRG scaffold. There is a drastic cell migration into the scaffold with higher concentration of PRG motif.

Spontaneous cell migrations were observed using the confocal microscopy 3-D image collections and reconstructions. These results in Fig. 8 showed the reconstruction images of PRG 10% (A) and of PRG 70% (B). In the case of cells in PRG 10% scaffold, the cells were attached on the surface of the peptide scaffold and did not penetrate into the scaffold (A2). However cells in the scaffold made of PRG 70%, the cells spontaneously migrated into the scaffold for ∼300 µm (B2). The migrated cells could be visualized clearly with the confocal imaging. The results demonstrate under otherwise identical culture medium conditions that higher concentration alone of PRG stimulated cell migration into the 3-D peptide nanofiber scaffold. This is a significant finding that a simple motif could have drastic influence on cell behaviors. It is much easier to produce the designer scaffold than to find complex and expensive soluble factors that show similar cell behavior. Again, these results are consistent with our previous observation of adult mouse neural stem cells that also migrate into the biologically functionalized 3D scaffolds.

## Discussion

### Fine control of designer peptide scaffolds

In this study, we selected three peptide motifs including osteogenic growth peptide derived motif (ALK: ALKRQGRTLYGF), Osteopontin cell adhesion motif (DGR: DGRGDSVAYG) and a biomimic designed two-unit RGD binding sequence (PRG: PRGDSGYRGDS) to directly extend the carboxyl termini of the self-assembling peptide AcN–RADARADARADARADA–CONH2 and obtain the designer self-assembling scaffolds. Although many more designer peptide scaffolds have been studied, these three showed the best results for bone cell activities. We thus studied them in further detail.

We examined not only the fine structures of the self-assembling peptide scaffolds comprising designer peptides and RADA16, but we also showed that functional self-assembling peptide scaffolds are readily formed by direct extension of the motif to self-assembling RADA16 peptide and through mixing these peptide scaffolds.

### Designer peptide scaffolds for bone cell studies

We showed that the designer scaffolds with biologically active motifs are better suited for bone cells than the pure RADA16 scaffold alone. This simple method will likely become very useful than those of polymer or natural collagen scaffold functionalization through chemical modification. Similar results were also obtained when study adult mouse neural stem cells using both pure and specifically designer scaffolds [15].

We then evaluated biological function of these designer peptides and pure RADA16 scaffolds for mouse pre-osteoblast MC3T3E1 cell proliferation. All three designer peptide scaffolds showed a significantly higher cell proliferation in comparison to the pure RADA16 scaffold, thereby suggesting that the designer peptide scaffolds promote pre-osteoblast proliferation.

We also evaluated the effect of the designer scaffolds for pre-osteoblast differentiation not only using alkaline phosphatase (ALP) activity as an early differentiation marker but also using secreted osteocalcin as a later differentiation marker relevant to biomineralization. ALK, DGR and PRG scaffolds showed both statistically significant ALP activity and osteocalcin concentration in comparison to pure RADA16 scaffold alone. Although the results in ALP activity and osteocalcin were correlated, the variations between scaffolds were larger in osteocalcin concentration than in ALP activity. This suggests that the effect of designer scaffolds appears more important in the later stage of differentiation.

The designer peptides DGR and PRG contain RGD cell attachment motif for integrin receptors. RGD sequence is known to promote osteoblast proliferation and differentiation[6], [14]. However PRG, which has two repetitive RGD sequences, has approximately five times higher osteocalcin secretion than DGR, which contains an RGD sequence.

For marrow stromal cells cultured on the polymer scaffold modified with an RGD sequence by linker polymer, others reported that the cell attachment was increased when the linker length was longer than the polymer cross-linking length[13]. In our current study of DGR peptide, the linker length between RGD sequence and RADA16 is 1.38 nm, whereas the distance between 2nd RGD sequence and RADA16 is 2.75 nm. The difference of linker length may influence the effectiveness of RGD sequence for cell attachment, and hence, differentiation. It has been reported that 3-D structure of RGD sequence in the scaffold may also affect the difference. Also linear RGD and cyclic RGD are known to stimulate different degrees of cell attachment, this is presumably given by the different 3-D structure of the two configurations[22], [23]. The difference may influence osteoblast cell attachment *in vitro* culture and bone formation *in vivo* [24].

### The concentration effect of designer peptide scaffolds

We evaluated the effect of the concentration of designer peptide containing 2 units of RGD (PRG). Our study showed that even 1% PRG∶99% RADA16 mixed scaffolds promote cell attachment, and proliferation. The cells were on the surface of the scaffold. On the other hand, when we used 40% PRG∶60% RADA16 and 70% PRG∶30% RADA16 mix ratios, cells not only spontaneously migrated into the scaffolds and retained the cell morphology but also showed an asteroid form. It has been suggested that different cell shapes showed different cell phenotype[25]. We demonstrated that the PRG peptide scaffold both encourages cell 3-D migration and promotes differentiation at the same time.

### Other advantages of designer peptide scaffolds

Previous studies show that scaffolds such as Demineralized Bone Matrix (DBM), collagen and alginate with solid porous scaffold composite composed with Hydroxyapatite, tricalcium-phosphate and polymer promote bone regeneration *in vitro* and *in vivo* [26]–[28]. However, these natural derived hydrogel biomaterials have several concerns for clinical use, 1) risk of infection agents from animals to human, 2) reproducible quality control, 3) limited shelf-life and 4) difficulty in effectively functionalizing the biomaterial[29]. Thus, attempts have been made to mimic the 3-D nanostructure of natural extracellular matrices with synthetic materials using electrospinning techniques[30], self-assembling peptide scaffolds and self-assembly of peptide chimerical amphiphilic nanofibers[31], [32].

Self-assembling peptide nanofiber scaffolds are biodegradable by a variety of proteases in the body with superior biocompatibility with the tissues (29). These self-assembling peptide scaffolds have an advantage in that they can be manufactured by a conventional, commercially chemical peptide synthesis methods and the cost is reducing steadily. This study further suggests that the second generation designer self-assembling peptide scaffolds may become superior designer biological functionality to nature derived extracellular matrices through molecular design of the scaffolds by direct addition of active and functional motifs selected from extracelluar matrices and cell secreted soluble peptides and proteins.

A wide range of designer, self-assembling peptide scaffolds for tissue regeneration in animal models is currently under evaluation in order to provide an effective strategy for regenerative medicine. The composite of the designer self-assembling peptides with solid porous scaffold including tricalcium-phosphate for specific bone regeneration are under further study.

### Conclusions

We have developed new biomimetic designer self-assembling peptide scaffolds to enhance pre-osteoblast proliferation, differentiation and migration. These designer self-assembling peptide scaffolds with mixing RADA16 showed remarkable effectiveness at enhancing cellular proliferation and differentiation activities. We selected three active motifs from cell secreted signal peptide (osteogenic growth peptide), call attachment domain of extracellular matrix (osteopontin) and designed RGD cell attachment sequence. This study clearly demonstrated that the designer self-assembling peptide scaffolds significantly enhanced mouse pre-osteoblast cell proliferation and differentiation as well as stimulating cell migration into the 3D scaffold.

Our study reported here has far reaching implications beyond the current study. The simple addition of short, biologically active peptide motifs can significantly enhance particular cellular activities, thus open a new avenue to design new biologically active scaffolds for a widespread use, not only for specific cell cultures, but also for specific tissue repair, tissue engineering and regeneration medicine.

## Materials and Methods

### Materials

RAD16 solution (1%) was purchased as PuraMatrix from BD Bioscience, Bedford, MA. These designer peptides were custom-synthesized (Synpep Corporation, CA and Genscript Corp. NJ). They were dissolved in water at a final concentration of 1% (w/v) and sonicated for 20 min (aquasonic, model 50 T, VWR, NJ). The designer peptide solutions were then mixed at a ratio of 1∶1 with 1% PuraMatrix solution, except otherwise stated. Each of the peptide solutions was directly loaded in the tissue culture plate inserts (10 mm diameter, Millicell-CM, Millipore, MA). The maintenance medium was added to induce hydrogel formation.

### Reagents

To maintain the MC3T3-E1 cell line, penicillin/streptomycin, minimal essential medium and FBS were obtained from Invitrogen Corp. (Carlsbad, CA, USA). Ascorbic acid and β-glycerophosphate were from Sigma Chemical Co. (St Louis, MO, USA). PicoGreen® dsDNA Quantitation kit (P-7589, Invitrogen, Eugen, OR) for DNA, Alkaline Phosphatase Fluorescence Assay Kit (Sigma, MO) for ALP activity and Mouse Osteocalcin EIA Kit (Biomedical Technologies Inc., MA) is used for Osteocalcin. The histological assays made use of Alkaline Phosphatase kit (85L-2, Sigma, MO) and calcein-AM staining (Live/Dead Viability kit L-3224, Molecular Probes, OR).

### Structural study using atomic force microscopy (AFM)

Peptide from stock solutions (0.5%) was diluted to a working concentration of 0.01% (w/v), after 30 min sonication, subsequent after 2 hours stationary incubation at room temperature. Atomic force microscopy (AFM) images were collected with a silicon scanning probe (FESP, Vecco Probe Inc., CA) with a resonance frequency of 75 KHz, spring constant 2.8 N/m, tip curvature radius 10 nm and 225 um length. Images were obtained with a Multimode AFM microscope (Nanoscope IIIa, Digital Instruments, CA) operating in Tapping Mode. Typical scanning parameters were as follows: tapping frequency 75 KHz, RMS amplitude before engage 1–1.2 V, set point 0.7–0.9 V, integral and proportional gains of 0.2–0.6 and 0.4–1.0 respectively, and scan rate 1.51 Hz.

### Preparation of MC3T3-E1 cells

Mouse pre-osteoblast cell line MC3T3-E1 (subclone 4) (ATCC, VA) was purchased for this study. Cells were maintained in the maintenance medium consisting of α modified essential medium with 10% FBS and 1.5% penicillin/streptomycin. The medium was changed every 3 days. When the cells became sub-confluent, they were detached from the flask by treatment with aqueous solution of 0.25% trypsin for 5 min at 37°C. The cells were normally sub-cultured at a density of 5×10^3^ cells/cm^2^.

### Cell culture system

The scaffolds 1.0% (w/v) were prepared as pure RADA16 or mixed with others at a radio of 1∶1 (v/v) (RADA16∶ALK, DGR or PRG (Table 1). Each solution was sonicated for 30 min and loaded (100 µl) on top of a cell culture insert (10 mm diameter, Millicell-CM, Millipore, MA) and allowed to form a layer ∼1 mm thick. The maintenance medium described later was gently added on the top of the scaffold to induce gelation.

The system was incubated at 37°C for 1 hour. Then the medium inside the insert was exchanged by the maintenance medium and the outside of the insert was filled with the maintenance medium and incubated for half a day within a cell culture incubator at 37°C with 5% CO_2_.

Cells were plated at 2×10^4^ cells on the hydrogels in the inserts. The cells were cultured in the maintenance medium Day 0 through Day 2 and then converted into the differentiation media supplemented with L-Ascorbic acid 50 µg/ml and glycerophosphate 10 mM. The media were changed every three days. The gel, cell lysis and culture medium were harvested at 14 days of culturing for analysis.

### DNA content measurement

The number of cells on the scaffold was determined by the fluorometric quantification of amount of cellular DNA. The cell-seeded scaffold was rinsed with PBS and recovered by Na Citrate buffer solution containing 50 mM Na Citrate and 100 mM NaCl and stored at −80° C until assay. After thawing, the cells were lysed in the Na Citrate solution with occasional mixing. The 10 µμl of cell lysate (400 µl/insert) was mixed with Na Citrate buffer (100 µl) and DNA binding fluorescent dye solution (0.5 µl Picogreen reagent in 100 µl 1XTE buffer). The fluorescent intensity of the mixed solution was measured on a fluorescence spectrometer (Wallace Victor2, 1420 Multi-label counter, Perkin-Elmer, MA, Ex 485 nm/Em 510 nm). The calibration curve between the DNA and cell number was prepared by use of cell suspensions with different cell densities.

### Biochemical assays for alkaline phosphatase (ALP) activity

ALP activity in the cells on hydrogel was determined by fluorometric quantification. The cell-seeded hydrogel was rinsed with saline and recovered by ALP lysis buffer containing 2 mM MgCl_2_ and 0.05% Triton X-100 and then stored with at −80°C until assay. After thawing, the cells were lysed in the ALP lysis solution with occasional mixing. The 20 µl of cell lysate (300 µl/insert) was mixed with 20 µl MgCl_2_ solution and 150 µl Fluorescent Assay Buffer both provided in Alkaline Phosphatase Fluorescence Assay Kit (Sigma, MO). Then 1 µl of suspended substrate (4-Methylumbelliferyl Phosphate, 10 mM) was added and incubation occurred at the room temperature for 1 hour. The fluorescent intensity of the mixed solution was measured on a fluorescence spectrometer (Wallace Victor-2, 1420 Multi-label counter, Perkin-Elmer, MA, Excitation 355 nm-Emission 460 nm). The calibration curve between the ALP activity and fluorescent was prepared by use of Alkaline Phosphatase (control enzyme) with different concentration.

### Biochemical assays for Osteocalcin

Osteocalcin was measured from cell culture medium using Mouse Osteocalcin EIA Kit (Biomedical Technologies Inc., MA) according to the manufacturer's protocol. Briefly, the medium was diluted by Milli-Q water×200; 25 µl of the diluted medium and 100 µl osteocalcin antiserum were placed in 96 well EIA plates and incubated at 4°C for 18–24 hours. The well was washed with PBS and 100 µl Streptavidin-Horseradish reagent was added and incubated for 30 minutes. 50 µl of TMB solution and Hydrogen Peroxide solution were added and incubated for 15 minutes at room temperature. After adding 100 µl stop solution, absorbance was measured at 405 nm on a colorimetric microplate reader (vmax kinetic microplate reader, Molecular Device, CA)

### Alkaline Phosphatase (ALP) staining

ALP staining was conducted using Alkaline Phosphatase Staining kit (85L-2, Sigma, MO) according to the manufacturer's protocol. Briefly, cells on the scaffold in the inserts were washed twice in saline, fixed for 6 min at room temperature in Citrate fixative solution contained in the kit, and then washed by Mill-Q water. AS-MX solution in the kit was added to the insert and incubated at room temp for 30 min. The insert was rinsed well by Mill-Q water for 3–4 times and the cells were observed under a light microscope.

### The effects of the mix ratio of functionalized and non-functionalize peptides

The effects of the mix ratio of functionalized peptide and non-modified peptide were studied using the PRG with the basic RADA16. A glycine was added to the C terminal (Ac-(RADA)4-GPRGDSGYRGDSG-CONH2) to PRG peptide. This modification makes PRG form the self-assembling hydrogel stably without disturbing biological function. The mix ratio between RADA16 and PRG was varied from 100∶0 (PRG 0%), 99∶1 (1% PRG), 90∶10 (10% PRG), 60∶40 (40% PRG), 30∶70 (70% PRG) up to 0∶100 (100% PRG). The other culturing conditions were as described above except that the cell was cultured for 7 days. The cell number was measured by the method described in the method section. The cell morphology on the hydrogel was examined using calcein-AM staining (Live/Dead Viability kit L-3224, Invitrogen, Eugene, OR) according to the manufacturer's protocol.

Briefly, cells on the peptide scaffolds of the inserts were washed twice using PBS. Then 4 µM calcein AM solution was added to the insert and incubated for 1 hour with in the incubator at 37°C with 5% CO_2_. The inserts were rinsed well by PBS for 2 times and the cells were examined under an optical microscope.

### Statistical analysis

All the data were statistically analyzed to express in the standard deviation (SD) of the mean. The t-test was performed and p<0.05 was accepted to be statistically significant.
